# Surgical Admissions and Treatment Outcomes at a Tertiary Hospital Intensive Care Unit in Ethiopia: A Two-Year Review

**DOI:** 10.4314/ejhs.v30i5.11

**Published:** 2020-09

**Authors:** Kirubel Abebe, Tesfaye Negasa, Fitsum Argaw

**Affiliations:** 1 Department of Surgery, St. Paul's Hospital Millennium Medical College; 2 Department of Surgery, Arsi University Asella School of Medicine and Health Sciences

**Keywords:** Intensive care unit, surgical admission, outcome

## Abstract

**Background:**

Intensive Care Unit (ICU) is a special unit where critically ill patients who require advanced respiratory or hemodynamic support are admitted. Little has been published about surgical intensive care unit patients in Ethiopia. The aim of this study was to assess the pattern of admission and treatment outcomes of adult surgical patients admitted to the Intensive Care Unit at St. Paul's Hospital Millennium Medical College (SPHMMC).

**Methods:**

A two-year retrospective medical record review of all adult surgical patients admitted to Intensive Care Unit at St. Paul's Hospital Millennium Medical College.

**Results:**

Surgical patients made up 91(22.1%) of 411 admissions of adult intensive care unit. Of these, 82 (M: F = 1.5:1) patients were analyzed. Age ranged from 16 to 82 years with a mean age of 43 years (SD +/-18.2). Emergency admissions accounted for 70(85.4%) cases. The top three primary admission diagnoses were generalized peritonitis secondary to perforated viscus (25,30.5%), bowel obstruction (21,25.6 %) and trauma (13,15.9%). Acute respiratory failure (38,46.3%) and septic shock (23,28.0%) were the leading indications of intensive care unit admission. Most patients (62,75.6%) received mechanical ventilatory support. The mean length of intensive care unit stay was 7.3 days (SD+/-5.2).Death occurred in 33(40.2%) patients. Mortality was higher in those who stayed for 48 hours (OR=5.6;95% CI 1.60–19.69; p=0.007) and in ventilated patients (OR=5.3; 95% CI 1.41–19.98; p=0.013).

**Conclusion:**

The observed mortality in this review was higher than the one in most reports. It was significantly high in patients who stayed for 48 hours and in those who required mechanical ventilatory support.

## Introduction

Intensive care unit (ICU) is a special unit, primarily concerned with the care of patients with critical illness (1–3).It demands broad-based knowledge, advanced monitors and organ support equipment to achieve good outcomes. The level of care and follow-up is more sophisticated and comprehensive than the one in the ordinary ward (1–10).

Identifying patients who would benefit from ICU admission is a major challenge in critical care medicine. Patients may die following denied ICU care due to admission of patients with poor prognosis. As a result, Intensive care is indicated for patients with potentially recoverable disease (1–3,8).

Recently, demand for surgical intensive care is increasing due a number of reasons such as advancement in operative procedures, improved critical care service and critical care technologies (5,11–13). Emergency department or general wards may act as a source for ICU admissions due to deterioration of patients' clinical condition or as a postoperative case from the operating theatre. Failure to awake from anesthesia, surgeries with high risk of complication and various sequalae of procedures are among the reasons of ICU care for a surgical patient.(8,11,14–17).

Critical care medicine is relatively new and at early stage in sub-Saharan countries including Ethiopia, despite the greatest burden of diseases which deserve ICU care (9,12,18). Reduced number of ICU beds, lack of sufficiently trained healthcare professionals, constraints in critical technologies, shortages of medications and scarce data on patients'outcome are the major challenges of the critical care in the developing nation (4,5,8,9,12,18).

Published data about surgical patients admitted to ICU is scarce in our country. To the best of our knowledge, a study done by Kinfu et al is the only published data on pattern of admission to surgical intensive care unit for mechanical ventilator (19). Conducting this review to assess the pattern of admission and treatment outcomes of adult surgical patients admitted to ICU will help to measure the magnitude of the problem, help to allocate and utilize ICU facility and to evaluate our surgical critical care.

## Method and Materials

Institution-based cross-sectional study design was employed from January 2016 to December 2017 to assess the pattern of admission and treatment outcomes of all adult surgical patients admitted to ICU at SPHMMC. SPHMMC is the second biggest hospital in Addis Ababa, Ethiopia, serving a catchment area of more than 6 million people. During the study period, the adult medical-surgical ICU had a capacity of 4 beds and was staffed with critical care nurses and physicians from different specialties. Basic Equipment such as mechanical ventilators, defibrillators, infusers, ultrasound, monitors and portable x-ray machines were available.

Neurosurgical (including head and spinal injuries) as well as orthopedic and plastic surgical patients were treated in a separate affiliation hospital (Addis Ababa Burn, Emergency and Trauma Center) and ICU. This study only includes those admitted to SPHMMC ICU. Therefore, the term “surgical patients/admissions” represents adult (age >14 years)general surgical cases, urologic and subjects with thoracoabdominal injury.

Medical Record Numbers (MRN) identified from the ICU registries and individual charts were retrieved from the hospital archive room. A total of 91 surgical patients were admitted to ICU during the study period and of these 82 patients were retrieved due to incomplete and lost medical records. Ethical clearance was obtained from the institutional review board, and data on patients' socio demographic characteristic, causes of ICU admission, length of ICU stay, ventilation status and patient outcome were collected by trained final year medical students using a pretested data collection format.

Data were checked for completeness, accuracy, consistency then coded and entered into SPSS version 23. Descriptive analysis was conducted to characterize the variables, and associations with outcome were identified and considered significant when p-value was <0.05. Results were presented in tables, charts and central tendency statistics.

## Results

Surgical patients accounted up 91(22.1%) of 411 admissions to adult ICU (Table 1). Of these, 82(90%)medical records were retrieved. Male constituted 49(59.8%) admissions with male to female ratio of 1.5:1.The mean and median age was 43 years (SD +/-18.2) and 45 years (16 to 82 years), respectively. Emergency patients constituted more than two third (70,85.4%) of the admissions.

**Table 1:** Pattern of admission to adult ICU at SPHMMC, Addis Ababa, Ethiopia from January 2016 to December 2017

Speciality/Department	Total n(%)
Internal medicine	227(55.2)
General Surgery	91(22.1)
Gyn/Obs	85(20.7)
Ear, Nose and Throat	8(2.0)

The top three primary diagnosis were peritonitis secondary to perforated viscus(25,30.5%), bowel obstruction (21,25.6%) and thoracoabdominal injury (13,15.9%). Acute respiratory failure (38,46.3%) and septic shock (23,28.0%) were the leading indications to ICU admission. Of these, septic shock (13/23,56.5%) and peritonitis secondary to perforated viscus (12/25,48.0%) had the highest case fatality rate. Most admissions (62,75.6%) received mechanical ventilatory support (Table 2).

**Table 2:** Primary diagnosis, Indications and case fatality rate of surgical patients admitted to ICU at SPHMMC Addis Ababa, Ethiopia from January 2016 to December 2017

Primary Diagnosis	Total n(%)	Mortality n(%)
Peritonitis due to perforated viscus	25(30.5)	12(48.0)
Bowel obstruction	21(25.6)	10(47.6)
Thoracoabdominal injury	13(15.9)	6(46.1)
Thyroid disease	6(7.3)	0(0.0)
Necrotizing fasciitis	5(6.1)	2(40.0)
Urosepsis (Pyonephrosis)	4(4.9)	1(25.0)
Gastrointestinal (GI) Cancer*	3(3.60	1(33.3)
Ascending cholangitis	3(3.6)	1(33.3)
Pancreatitis	2(1.2)	0(0.0)
**Indications of ICU admission**		
Acute respiratory failure	38(46.3)	16(42.1)
Septic shock	23(28.0)	13(56.5)
Anaesthesia related incidents	11(13.4)	3(36.4)
Upper Airway Obstruction (post thyroidectomy)	6(7.3)	0(0.0)
Pulmonary thromboembolism	4(4.9)	1(25.0)

* Gastric and colonic cancers

Regarding length of ICU stay, the mean was 7.3 days (SD+/-5.2), and the median was 6 days (12 hours to 28 days). The most frequent (29,35.4%) stay was between 3 to 7 days, and 6 (7.3%) patients stayed for less than 24 hours. Mortality was higher in those who stayed for 48 hrs (11/15,73.3%) (Table 3).

**Table 3:** Length of ICU stay and outcome of surgical patients admitted to ICU of SPHMMC, Addis Ababa, Ethiopia from January 2016 to December 2017

Length of ICU stay (days)	Total n (%)	Mortality n (%)
<1	6(7.3)	6(100)
1–2	9(11.0)	5(55.5)
3–7	29(35.4)	10(34.5)
8–14	27(32.9)	8(29.6)
>14	11(13.4)	4(36.4)

Of the admissions, 33(33/82,40.2%) patients died in the ICU while 5(5/49,10.2%) patients died in the ward after ICU discharge. The overall mortality of critically ill surgical patients in the hospital was 46.3% (38). The most common cause was multi-organs failure (MOF) (22,66.7%) followed by cardiac arrest (6,18.2%) and respiratory failure (5,15.1%). The highest mortality rate (9/15, 60%) was noted in the age group of 55 to 64 years (Table 4).

**Table 4:** Mortality rate and age groups of surgical patients admitted to ICU of SPHMMC, Addis Ababa, Ethiopia from January 2016 to December 2017

Age in years	Total, n (%)	Mortality n (%)
15–24	14(17.1)	4(28.6)
25–34	19(23.2)	5(26.3)
35–44	9(11.0)	4(44.4)
45–54	12(14.6)	6(50.0)
55–64	15(18.3)	9(60.0)
65–74	10(12.2)	5(50.0)
75–84	3(3.7)	0(0.0)

**Associated factors with mortality**: Mortality was increased in those who stayed for 48 hours (OR=5.6;95% CI 1.60–19.69; p=0.007) and in ventilated patients (OR=5.3; 95% CI 1.41–19.98; p=0.013) than their respective groups. Older age, sex, mode and indication of admission had no statistically significant associations with mortality (Table 5).

**Table 5:** Factors affecting mortality of surgical patients admitted to ICU at SPHMMC Addis Ababa, Ethiopia from January 2016 to December 2017

Variables	Outcome (mortality)		OR for mortality	P value

	Died	Improved		
**Age**				
<55	19	35	1	0.197
>=55	14	14	0.54(0.21–1.37)	
**Sex**				
Male	21	28	1.31(0.53–3.25)	0.577
Female	12	21	1	
**Mode of admission**				
Emergency	31	39	0.25(0.05–1.23)	0.089
Elective	2	10	1	
**Use of ventilator**				
Ventilated	30	32	5.3(1.41–19.98)	0.013*
Not ventilated	3	17	1	
**Indication for ICU admission**				
Acute respiratory failure	16	22	0.87(3.57–2.10)	
No acute respiratory failure	17	27	1	0.749
Septic shock	13	10	0.39(0.15–1.06)	0.064
No septic shock	20	39	1	
**Length of ICU stay**				
<=48 hrs	11	4	5.62 (1.60–19.69)	0.007*
>48 hrs	22	45	1	

* Statistically significant at P<0.05, OR: Odds Ratio

## Discussion

Worldwide, the demand for surgical intensive care is emerging for reasons of recent advances in operative interventions with improved critical care service and critical care technologies (5,11–13).The reported burden of surgical intensive care admissions varies from 15.3% to 86.8% (5–7, 9–11,13,16,19–24). Towey et al described the ICU in the sub-Saharan Africa as a surgical ICU because postoperative surgical patients made the highest admissions (9).On the contrary, our analysis and a report from another ICU in Ethiopia done by Smith et al demonstrated medical patients as the main group of ICU admissions (22). Audits of Intensive Care Over Nations (ICON) and studies of Nepal, Jordan and Ireland parallel the finding of this study (10,11,13,21). The possible reasons for the discrepancy include variation in disease prevalence, ICU organization and availability of trained staffs. Even if the burden in this study was within the reported range, it is much lower than most studies. Our study excluded neurosurgical (including head and spinal injuries), burn, polytrauma and orthopedic patients due to a separate ICU and treatment center at an affiliation hospital. The absence of subspecialties like cardiothoracic and vascular surgery in our hospital could also be the reason for the lower rate. On the other hand, some studies included obstetric/gynecologic cases as a surgical patient which contributed for the higher surgical ICU admission rate (5–7,10,19,20,22). If this group of patients are excluded, the surgical ICU admission percentage of our series will be comparable with findings of smith el al (28.6%) and Onyekwulu et al (20.5%) (20,22).

In our analysis and others', males and emergency surgeries made the majority of surgical ICU admissions (5,7,11,13,15–17,24,25). This could be due the higher disease prevalence like trauma in males and lack of adequate time for preoperative optimization in emergency patients. The mean age ranges from 29 to 62 years which agrees with this study (5,7, 16,18,21,24,25). Smith et al reported a median age of 32 years at Jimma University Specialized Hospital (JUSH), Ethiopia, which is younger than our study (45 years). This could be due to the higher trauma prevalence in young patients; hence, trauma was the top primary diagnosis in their study (22). The higher life expectancy in developed nations may contribute to the increased mean age of critical patients in USA (57.6 years) and Europe (61.5 years) (21,25).

Most of our subjects underwent abdominal surgery for complicated acute abdomen and thoracoabdominal injury. This is consistent with findings of similar studies from Africa, Europe and USA (6,7,11,20,25). In contrast, other studies identified trauma specifically head injury as the major cause of surgical critical care admission (5,10,13,15,16,18,19,22,24). Differences in study subjects, disease prevalence and ICU setup could explain this discrepancy. In this review, thyroidectomy for thyroid pathologies constituted higher number than the one reported elsewhere in Ethiopia (7.3% vs 3.2%) (22). This could be due to variation in burden of the diseases provided that it is the leading elective surgical admission and procedure in our hospital (26). The burden of acute soft tissue infection among our subjects was similar with reports of Smith et al (22).

Septic shock and acute respiratory failure were the leading immediate indications of ICU admissions in this study and other studies (5,17,18,21). Some authors advocate planned admission for high risk surgical patients since it accounts for 80% of all perioperative deaths (14,17). Post-operative monitoring or observation is also among the top indications for surgical ICU admission as it is mentioned by literatures (16,18,23,24). In our review, anesthesia related incidents made up 13.4% of all surgical ICU admissions which is higher than findings of Prin M et al (1.8%) and Onyekwulu FA et al (1.5%) (16,20). This may be attributed to availability of well-trained staff, functioning equipment and essential medications (20). On the other hand, proper preoperative evaluation and patient factors may also contribute to this difference.

As compared to other studies, our patients stayed longer in the ICU (11,15,22,24). There are many possible reasons for this, including difference in study subjects, presence of high dependence/intermediate unit and proper ICU admission criteria. Most (74.3%) of our study subjects had acute respiratory failure and septic shock due to intraabdominal sepsis that deserve more invasive procedures and longer follow-up than trauma cases. Lissauer et al reported that the mean ICU length of stay ranged from 4.47 days for neurosurgical patients to 13.5 days for acute emergency surgical patients. Many of their patients transferred for problems of inability to be handled at local hospitals that require advanced and long-term care (25).

Most authors reported that 57.7%–80% of all ICU admissions required mechanical ventilatory support (5,10,18–20,25,18). An Ethiopian study done by Kinfu et al reveled 60.7% ventilatory support in the ICU utilized by surgical patients (19). In line with this, our review and a report from USA demonstrated that almost two-thirds of surgical admissions were put on mechanical ventilator (25). On the contrary, studies from Nigeria showed very low percentages(15% and 19%) of ventilatory support (7,16). The number of mechanical ventilators available and the indication for admission may attribute to this variation.

In this analysis, the in-ICU mortality rate for surgical patients was 40.2% which is in the reported range (15.1% in USA to 48% Ethiopia) by different authors (6,7,10,15–17,20,22,25). This discrepancy depends on factors such as the case mix, ICU setup, severity of the illness, patient factors (co-morbidity, delay in presentation and age), length of ICU stay and the care prior to ICU admission (5,16,21,22). The study also demonstrated a significant number of deaths among patients who spent 48 hours (OR 5.6, P<0.05). This finding is in line with a review by Onyekwulu FA et al (16). Apart from the severity of the illness, this may signify inappropriate or delayed admission to the ICU. Targeted admission criteria for those who benefit most is crucial for proper utilization of ICU (2,3,16,20). As it is observed in our study and others', emergency abdominal surgeries were associated with high case fatality rate (CFR) (6,7,10,15,17,24,25). Reports from Nigeria, Malawi and Tanzania demonstrated high CFR in peritonitis and intestinal obstruction which is also true in our analysis (6,7,24). Of the indications for ICU admission, septic shock had the highest mortality rate as it is seen in our and other studies (9,17,18,20,21,23). Even in best setups, death from septic shock may reach up 40 to 50% (20,23). This is worse in in low- and middle-income countries (21). Despite higher number of admissions due to anesthesia related incidents, the mortality rate was lower than a Malawi study (36.4 % Vs66.7%) and higher than the one in developed nations (20,27). This could reflect differences in quality of anesthesia care provided and patient factors. In contrast to our study, a previous Ethiopian study found high mortality rate (58.3%) in acute soft tissue infections. The reason may be due to inclusion of surgical site infections as a soft tissue infection unlike our review. In this review, ventilated patients had higher chance of death (OR 5.3, P<0.05) than the respective group. In agreement with this, a study in sub-Saharan African countries revealed 50% of chance of mortality in artificially ventilated patients which is seen also in Malawi and Nigeria studies (6,9,16). The in-hospital mortality rate after discharge from ICU was higher in our review than the one found by VDMayr et al (4.3%) (23). This may reflect the absence of high dependence/intermediate unit (HDU) in our hospital where patient-to-nurse ratio and follow-up were better than the ones in surgical wards.

In general, our study demonstrated the situation of surgical critical care service in a resource limited setting. The majority of our patients presented with intra-abdominal conditions such as perforated viscus, bowel obstruction and trauma complicated with septic shock and respiratory failure. The observed mortality in this review was higher than most reports. It was significantly increased in patients who stayed for 48 hours and in those who required mechanical ventilatory support.

The major limitation of this study is failure to assess the whole surgical ICU care since it does not include patients from other surgical disciplines including neurosurgical, cardiothoracic, orthopedic, etc. It also lacks data on scoring of disease severity, comorbid illness, duration of surgery, blood transfusion, use of inotropes and details of mechanical ventilatory support which helps to evaluate the capacity of ICU utilization and patient's outcome. Despite these drawbacks, it provided a baseline data about surgical ICU care in resource limited setting that could help for decision making.

We recommend measures like setting appropriate and targeted criteria for ICU admission, establishing HDUs and improving anesthesia care could help in reducing burden and deaths in the care of our critical surgical patients. Provision of proper chart keeping and computerized documentation can be considered in maintaining accuracy and retrieving relevant data. Moreover, detailed, large and multi-centric studis need to be conducted for more conclusion and further result.
